# Role of MCM2–7 protein phosphorylation in human cancer cells

**DOI:** 10.1186/s13578-018-0242-2

**Published:** 2018-07-24

**Authors:** Liangru Fei, Hongtao Xu

**Affiliations:** Department of Pathology, College of Basic Medical Sciences and the First Affiliated Hospital, China Medical University, No.77 Puhe Road, Shenyang North New Area, Shenyang, 110122 Liaoning Province People’s Republic of China

**Keywords:** MCM, Phosphorylation, DNA replication, Checkpoint response, Cell cycle

## Abstract

A heterohexameric complex composed of minichromosome maintenance protein 2–7 (MCM2–7), which acts as a key replicative enzyme in eukaryotes, is crucial for initiating DNA synthesis only once per cell cycle. The MCM complex remains inactive through the G1 phase, until the S phase, when it is activated to initiate replication. During the transition from the G1 to S phase, the MCM undergoes multisite phosphorylation, an important change that promotes subsequent assembly of other replisome members. Phosphorylation is crucial for the regulation of MCM activity and function. MCMs can be phosphorylated by multiple kinases and these phosphorylation events are involved not only in DNA replication but also cell cycle progression and checkpoint response. Dysfunctional phosphorylation of MCMs appears to correlate with the occurrence and development of cancers. In this review, we summarize the currently available data regarding the regulatory mechanisms and functional consequences of MCM phosphorylation and seek the probability that protein kinase inhibitor can be used therapeutically to target MCM phosphorylation in cancer.

## Background

DNA is replicated via a multi-protein machinery comprising DNA polymerase, helicase, primase, circular sliding clamps, a pentameric clamp loader, single-strand binding protein (SSB) and other components [1–5]. This machinery is often referred to as a “replisome”. Initiation of DNA replication in each cell cycle is fundamental to maintain genomic integrity and stability. Key to initiation is the formation of pre-replicative complexes (pre-RCs) in late M/early G1 phase through the recruitment of MCM2–7 in an origin recognition complex (ORC)-, Cdc6-, and Cdt1-dependent manner [6–9]. After this key step, Dbf4-dependent kinase (DDK) and cyclin-dependent kinases (Cdks) phosphorylate MCM2–7, leading to the recruitment of Cdc45 and GINS (Go, Ichi, Ni, and San) to form the CMG (Cdc45–MCMs–GINS) replicative helicase complex. The CMG replicative helicase complex has a robust helicase activity [10–13]. In addition, emerging studies suggest that MCM2–7 plays a critical role not only in replication, but also in transcription [14, 15], replication checkpoint [16–18], and RNA splicing [19]. As MCMs also belong to the ATPases associated with diverse cellular activities (AAA+) family, they display ATPase activity [20]. Moreover, owing to the crucial function of MCMs, the regulatory mechanisms that modulate and control its activity are diverse and complex, particularly, the phosphorylation mechanism.

Multiple phosphorylation sites were distributed on the MCM2–7 subunits. The biological and functional consequence of MCM phosphorylation appears to be correlated with specific kinases and their phosphosites. Some MCM subunits undergo dynamic phosphorylation in a cell cycle-specific manner, which may be consistent with their cell-cycle-specific functions [21–25]. Aberrant phosphorylation of MCMs disrupts DNA replication and cell cycle progression, leading to diseases or cancers [26–31]. Several reviews have been published on MCMs. However, few specifically discuss the role of phosphorylation on MCM function. Here, we highlight the function and mechanism of MCM2–7 protein phosphorylation in human cancer cells.

## Phosphorylation of MCMs by Cdc7

Cell division cycle 7 (Cdc7) is an evolutionary conserved serine-threonine kinase that promotes the initiation of DNA replication by targeting the functional substrate MCM2–7 protein [32–35]. Similar to Cdk, Cdc7 is activated by its regulatory subunits: Dbf4 and Drf1 in human [36, 37]. Cdc7 is found to be up-regulated in various cancers and has been characterized as an independent prognostic marker and a potential therapeutic target [38–41].

Cdc7 preferentially phosphorylates MCM2 as well as other MCM subunits (Table 1). Although there is agreement regarding specific phosphosites, each study has also identified additional sites. Differences in cell line, experimental design, or detection sensitivity may contribute to inconsistency of results among studies. In general, Cdc7 phosphorylation of MCMs is essential for the initiation of DNA replication. Tsuji et al. identified three Cdc7-dependent MCM2 phosphosites (Ser-27/41/139), both in vivo and in vitro [21]. A triple alanine substitution at these three sites in MCM2 did not support DNA replication in HeLa cells. This suggests that Cdc7 phosphorylation of MCM2 was essential for the initiation of DNA replication. In addition, this study revealed that MCM2 accumulated on chromatin early in the G1 phase before Cdc7 phosphorylation during the G1/S phase. Phosphorylation of MCM2 did not affect the chromatin loading of MCM complex. However, another study by Chuang et al. suggested that Cdc7 phosphorylated MCM2 at Ser-5 prior to chromatin loading. As a result, MCM2, along with other MCM subunits accumulates with the chromatin during cell cycle re-entry [42]. However, both of the research groups concurred that Cdc7 phosphorylation of MCM2 had no effect on MCM complex formation [21, 42]. The difference between studies may indicate that biological and functional consequences of MCM2 phosphorylation by Cdc7 is regulated in a phosphosite-dependent manner. This finding is consistent with a study by Montagnoli et al. In this study, the authors demonstrated that Cdc7 phosphorylation of MCM2 isoforms showed different a affinity for chromatin, although their variable properties were similar during the cell cycle [24]. In addition, this study identified seven phosphosites in the N-terminus of MCM2 by Cdc7 (Ser-40/53/108), Cdk1/Cdk2 (Ser-13/27/41), and CK2 (Ser-139) in vitro. In cells, the MCM2 protein was phosphorylated on all of these sites. However, only Ser-40/53/108 was Cdc7-dependent in vivo. In non-synchronized cells, pSer-53 MCM2 was detected both in the soluble and chromatin-enriched fractions, whereas phosphorylated MCM2 at Ser-40 and Ser-108 was only detected in the soluble fractions. However, in cells homogeneously arrested in S-phase by hydroxyurea (HU), pSer-108 and pSer-40 MCM2 were detected in chromatin-associated fractions. In addition, Ser-108 has also been reported as an ataxia-telangiectasia-mutated (ATM)/ATM- and Rad3-related (ATR) kinase phosphosite on MCM2 [43, 44]. This may reflect an overlapping regulatory function between Cdc7 and ATM/ATR in replication fork machinery under replication stress. Certain studies [45–47] also confirm this phenomenon. Furthermore, Montagnoli et al. demonstrated that MCM2 phosphorylation at Ser-41 (putative CDK-dependent site) and Ser-139 (putative CK2-dependent site) were not affected by reducing Cdc7 [24]. In the study by Tsuji et al., the two sites were Cdc7-dependent phosphosites [21]. This may imply that multiple kinases were involved in N-terminal phosphorylation of multiple sites in MCM2. However, results obtained from the two groups suggested that Ser-40 and Ser-108 in MCM2 were Cdc7-dependent phosphosites, both in vivo and in vitro [48]. However, functional consequences of the two phosphosites remained unclear in their study. In contrast to MCM2, phosphorylation of MCM4 remains less-studied. Masai et al. demonstrated that Cdc7-mediated N-terminal phosphorylation of MCM4 contributes to initiation of DNA replication and cell growth by promoting chromatin loading of Cdc45, a key replisome member [22]. Furthermore, N-terminal phosphorylation of MCM2, MCM4, and MCM6 might be redundant, but esssential in the initiation of DNA replication. Because the combination of MCM2, -4, and -6N-terminal mutations resulted in the loss of cell viability, these mutations alone did not affect DNA replication or growth [22]. MCM subunits have similar function in replication regulation, but they are not identical. A recent study revealed that Cdc7/Dbf4-dependent hyperphosphorylation of MCM4, but not MCM2, correlates with replication initiation [49]. Thus, a majority of MCMs phosphosites undergo dynamic phosphorylation mediated by Cdc7 in a cell-cycle-specific manner. This phosphorylation pattern is consistent with the role of MCM2–7 in the initiation of DNA replication, which confirms that genomic duplication occurs once per cell cycle.

**Table 1:** Summary of MCM phosphorylation in human cancer cells

Protein	Kinase	Phosphorylation site or domain	Biological and functional significance	References
MCM2	Cdc7	S27, S41, S139	Suggested to be essential for initiation of DNA replication and ATPase activity of MCM complex	[16]
	Cdc7	S5	Promotes pre-RC assembly during cell cycle re-entry	[37]
	Cdc7	S40, S53, S108	Regulates MCM2’s chromatin loading in a site-dependent manner	[19]
	Cdc7	S40, S108	Unclear	[43]
MCM4	Cdc7	N-terminal	Promotes MCM4’s interaction with Cdc45 on chromatin. Suggested to be essential for initiation of DNA replication and cell growth	[17]
MCM3	Cdk2/CycE	T722	Promotes MCM3’s chromatin loading. Regulates S phase checkpoint activation	[12]
MCM4	Cdk2	S3, T7, S32, S54, T110	Decreases chromatin loading of MCM complex to avoid re-replication during mitosis	[45]
	Cdk1, Cdk2	S3, T7, T19, S32, S54, S88, T110	Inhibits chromatin loading and helicase activity of MCM complex. Blocks DNA replication through inactivation of MCM complex	[20, 46–49, 51]
MCM7	Cdk2/CycE, Cdk1/CycB	S121	Promotes MCM complex formation. Regulates S checkpoint activation and mitotic exit	[13]
MCM2	ATR	S108	Responds to replication stress and stabilizes replication forks	[39]
	ATR	S108	Responds to replication stress	[38]
MCM3	ATM	S535	Responds to replication stress	[38]
	ATM, ATR	S728	Responds to replication stress	[58]
MCM6	ATR	S13	Responds to replication stress	[59]
MCM2	SIK1	Unclear	Activates helicase activity of MCM complex during DNA replication	[61]
MCM3	DAPK	S160	Unclear	[62]
MCM4	EBV-PK	T19, T110	Blocks DNA replication through inactivation of DNA unwinding by the MCM4/6/7 complex. Leads to cell growth arrest	[26]
MCM7	p56^Lyn^	Tyr600	Promotes MCM complex formation, chromatin loading, DNA synthesis and cancer cell proliferation. Pathologically correlates with poor survival of breast cancer patients	[23]
	Akt	Unclear	Increases MCM7’s chromatin loading and MCM complex formation. Promotes DNA replication and cancer cell proliferation	[24]
	ILK	Unclear	Inhibits MCM7’s chromatin loading and cancer cell growth	[25]

## Phosphorylation of MCMs by Cdk

Cell cycle progression through each phase is tightly regulated by Cdks and their regulatory proteins, cyclins. Alterations in these proteins, lead to uncontrolled cell division, which is a characteristic of many cancers. Several lines of evidences have shown that MCMs are substrates of Cdks and some Cdk-dependent phosphosites on MCMs have been identified and characterized (Table 1). Moreover, a few phosphosites are stimulated by Cdk and Cdc7 simultaneously [22], indicating the functional crosstalk between the two classes of kinases. In general, Cdk-mediated phosphorylation of MCMs preferentially contributes to the cell cycle regulation. A study by Li et al. [17] revealed that Cdk2/CycE phosphorylation of MCM3 Thr-722 promoted its chromatin loading. Excessive MCM3 chromatin loading activated the checkpoint pathway, which as a result blocked the S phase entry, but did not affect mitotic exit. Similarly, phosphorylation of MCM7 also affects its function in cell cycle procession. In a study conducted by Wei et al. [18], it was found that both Cdk2/CycE and Cdk1/CycB phosphorylate MCM7 at Ser-121. Moreover, overexpression of the wild type (WT) MCM7, but not the MCM7-S121 mutant, resulted in an S phase block. This then activates the checkpoint kinase 1 (Chk1) checkpoint pathway through single-stranded DNA (ssDNA) accumulation in a p53-dependent manner. Phosphorylation of MCM7 at Ser-121 also contributes to the formation of MCM complex for a proper mitotic exit. Taken together, Cdk phosphorylation of MCM7 plays an important role in both activation of S phase checkpoint and regulation of proper M phase progression. Besides, the MCM7-S121 mutant, but not wild type MCM7, binds more efficiently to the chromatin [18]. This may indicate that the phosphorylation of MCM7 hinders its loading onto DNA. Similarly, Cdk1/2-dependent phosphorylation of MCM4 at multiple sites decreases the binding of MCM complex to DNA, avoiding re-replication during mitosis [50, 51]. Therefore, phosphorylation of MCM7 at Ser-121 may also contribute to proper mitotic exit by inhibiting the MCM complex association with chromatin. Additionally, MCM4 phosphorylation not only induced the MCM complex release from chromatin, but also inactivated the complex. Previous studies indicated that MCM4 phosphorylation at specific sites leads to loss of subassembly MCM4/6/7 DNA helicase activity [52, 53], which is necessary for initiating replication [54, 55]. Cdk-mediated phosphorylation of MCM4 may also be a critical checkpoint in the cell cycle because MCM4 phosphorylation levels at several Cdk sites, stimulated by HU or ultraviolet (UV) irradiation, correlated inversely with the level of DNA synthesis to some extent [56]. In addition to Cdk, ATR-Chk1 is also involved in this phosphorylation [56]. Another study by Komamura-Kohno demonstrated that some Cdk-dependent sites of MCM4 were differentially phosphorylated during the cell cycle [25], indicating MCM4 phosphorylation may have several distinct and site-specific roles. Interestingly, studies on the nuclear localization of chromatin-bound MCM4, phosphorylated at the Ser-3 and Ser-32 by Cdk2, showed that the nuclear localization was not generally colocalized with replicating DNA [25]. Similarly, Tsuji et al. have also reported that chromatin-bound Cdc7/Dbf4 phosphorylated MCM2 did not co-localize with replication foci during G1/S and S phase [21]. One explanation for this discrepancy is that MCM helicase work at a distance from replication forks as a rotary motor that pumps DNA along its helical axis by simple rotation [57, 58].

## Phosphorylation of MCMs by ATM/ATR

Replication stress, commonly known as the slowing down or stalling of replication forks, is a major source of mutations that contribute to genomic instability and tumorigenesis [59, 60]. This threat triggers a DNA damage response (DDR). ATM and ATR, the master regulators of DDR, phosphorylate substrates to stabilize the DNA replication fork and activate cell cycle checkpoints. The checkpoint signaling pathways slow cell cycle progression, thus allowing the cell to recover and prevent inappropriate entry into mitosis [61, 62]. Several studies have suggested that ATM/ATR-dependent checkpoint pathways are directly linked to the subunits of the MCM complex (Table 1). It was suggested by Shi et al. [63] that ATM phosphorylated human MCM3 C-terminal at Ser-728, in response to DNA damage. The MCM3 phosphorylated form was preferentially localized to the nucleoplasmic fraction, and the phosphorylation did not alter its association with chromatin and other MCM subunits. One interpretation of this finding is that the chromatin-bound MCM3 C-terminal is inaccessible to ATM because of special structural changes, which may allow chromatin-bound MCM3 to escape checkpoint-mediated inhibition or may prevent chromatin loading of nucleoplasmic MCM3 during DNA damage [63]. ATR also contributed to MCM3 C-terminal phosphorylation in response to DNA replication stress. However, ATM still accounts for a major part of phosphorylation [63]. Therefore, ATM and ATR may contribute differently to MCM subunits phosphorylation under replication stress. Also, it has been reported that ATM phosphorylated MCM3 at Ser-535 in response to ionizing radiation (IR) [43]. Whereas ATR phosphorylated MCM2 at Ser-108 in response to multiple forms of DNA damage and stalling of replication forks including: IR, UV light, HU, and polyamides [43, 44]. In contrast to most ATM/ATR substrates, pSer-108 MCM2 was detected in the absence of exogenous DNA damage [43]. This corresponds with other reports on MCM2 phosphorylated at Ser-108 by Cdc7 [24, 48, 49]. In a recent study, Ser-13 at MCM6 was also reported to be a novel putative ATR target site in response to replication stress [64]. The identification of these ATM/ATR-dependent phosphosites suggests that MCM phosphorylation may be required for cells to repair DNA damage, restart replication, and recover from arrest coordinating with the checkpoint under replication stress. In concordance with this hypothesis, Izawa et al. reported that HECT and RLD domain containing E3 ubiquitin protein ligase 2 (HERC2), an E3 ligase critical for DNA damage repair pathways, regulates DNA replication progression and origin firing by facilitating MCM2 phosphorylation under replication stress [65].

## Phosphorylation of MCMs by other kinases

Other than the three classes of kinases Cdc7, Cdk, and ATM/ATR, several additional kinases were also shown to be involved in MCM phosphorylation (Table 1). Among these, p56^Lyn^-, Akt-, and integrin-linked kinase (ILK)-mediated MCM7 phosphorylation primarily correlates with the development of human cancer. MCM7 phosphorylation at Tyr-600 mediated by epidermal growth factor receptor (EGFR)-p56^Lyn^-axis promotes MCM complex assembly and chromatin loading, consequently enhancing DNA synthesis and cancer cell proliferation [28]. Furthermore, the Tyr-600 phosphorylation of MCM7 correlates with poor survival of breast cancer patients [28]. Similarly, Akt-dependent phosphorylation of MCM7, mediated by receptor for activated C kinase 1 (RACK1), also facilitates association of MCM7 with chromatin and MCM complex formation. As a result, this promotes DNA replication and cell proliferation in non-small cell lung cancer [29]. In contrast to these oncogenic roles, MCM7 phosphorylation mediated by the integrin ɑ7 (ITGA7)-ILK axis reduces MCM7 chromatin association thus inhibiting cell growth. This suggests that phosphorylation may be a critical event in the ITGA7 tumor suppression signaling pathway [30]. However, specific Akt-dependent and ILK-dependent phosphosites for MCM7 remain unclear. Phosphosites of MCM7 and the signaling pathways involved may affect the role of MCM7 phosphorylation in cancer development. In addition to MCM7, MCM2 was shown to be a substrate of salt-induciblekinase1 (SIK1) [66], MCM3 was reported to be phosphorylated by death-associated protein kinase (DAPK) at Ser-160 [67], and MCM4 was phosphorylated by Epstein–Barr virus-encoded protein kinase (EBV-PK) at Thr-19 and Thr-110 [31]. Among these, SIK1-dependent MCM2 phosphorylation, mediated by Sld5, is required for MCM helicase activity, but it does not affect the chromatin association of MCM2 [66]. The authors identified five SIK1-dependent phosphosites on MCM2 in vitro; however, specific phosphosites in cells need further identification. In HeLa cells, EBV-PK phosphorylates MCM4 and shares at least two of the same sites (Thr-19 and Thr-110) with Cdk2. This results in the loss of MCM4/6/7 subassembly’s enzyme activity, which leads to cell growth arrest [31]. In addition, most of the pThr-110 MCM4 detaches from chromatin; however, about half of pThr-19 MCM4 is bound to chromatin [31]. However, the overall chromatin-bound MCM did not change, indicating that other mechanisms may be involved. Besides, EBV-PK might also phosphorylate MCM6 and additional sites of MCM4 to block DNA replication in Epstein–Barr virus (EBV)-infected cells [31]. In contrast to the MCM2 and MCM4 phosphorylation, the consequences of DAPK-mediated MCM3 phosphorylation remains to be elucidated [67].

## Conclusions

**Fig. 1:**
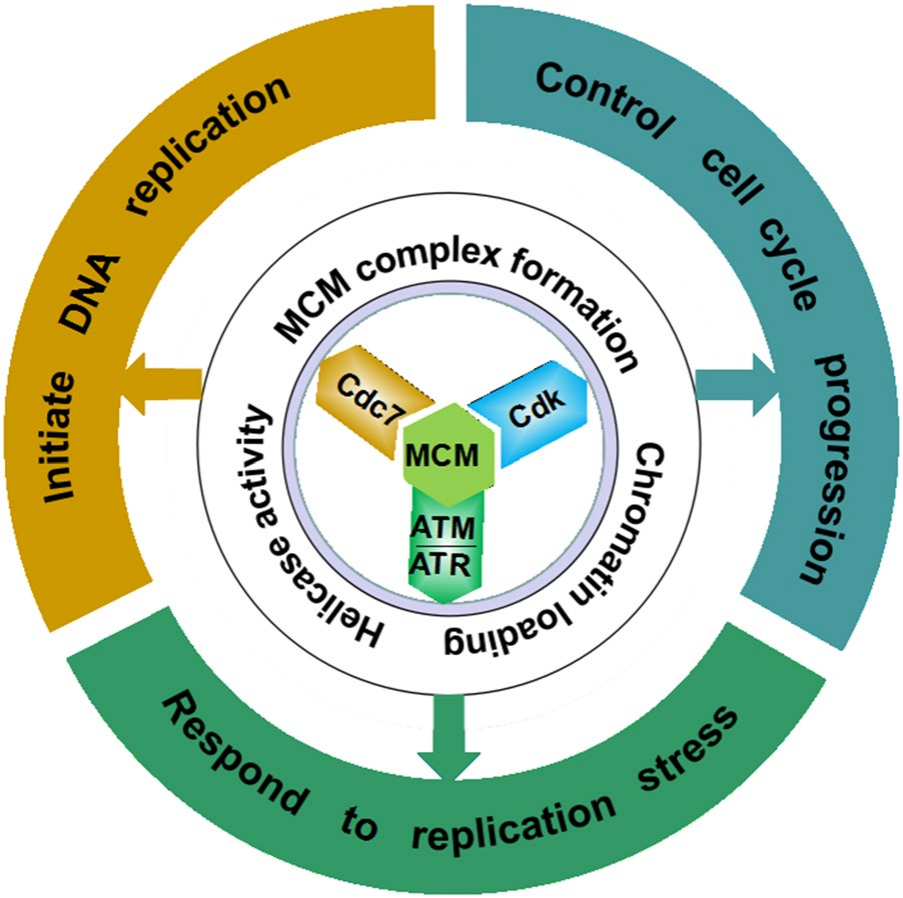
Schematic diagram summarizing the roles of MCM phosphorylation mediated by three classes of kinases Cdc7, Cdk and ATM/ATR. Although many functional crosstalks exist between MCM kinases-mediated phosphorylation events, evidence shows that Cdc7-dependent MCM phosphorylation primarily promotes the initiation of DNA replication, Cdk-dependent MCM phosphorylation mainly contributes to cell cycle progression regulation, and ATM/ATR-dependent MCM phosphorylation primarily responds to replication stress. MCM phosphorylation contributes to these various functions primarily by affecting MCM complex formation, chromatin binding, and (or) helicase activity

**Fig. 2:**
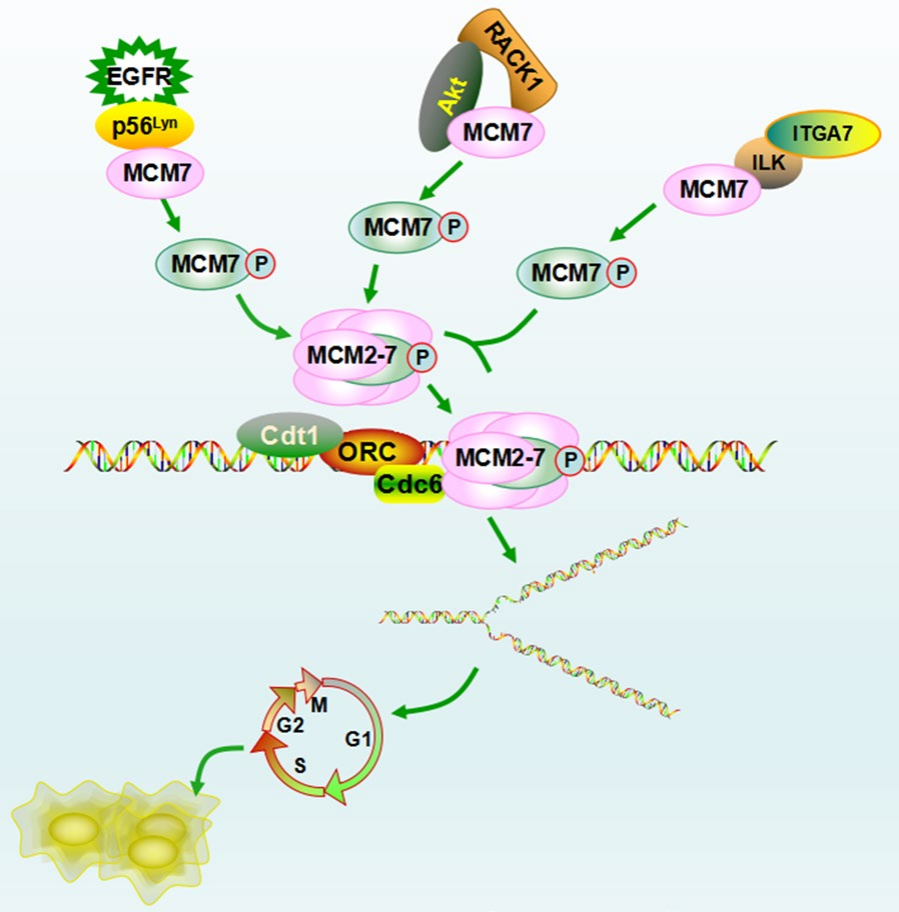
Roles of MCM phosphorylation mediated by p56Lyn, Akt and ILK in cancer development. Phosphorylation of MCM7 mediated by EGFR-p56^Lyn^ and RACK1-Akt promotes MCM complex assembly and chromatin loading, therefore enhancing DNA synthesis and cancer cell proliferation. In contrast, MCM7 phosphorylation mediated by ITGA7-ILK axis reduces MCM7 chromatin association, inhibiting cell growth

MCM proteins are regulated by multiple kinases and the consequences of these phosphorylation events reflect the critical integration of DNA replication with cell cycle and the checkpoint response (Fig. 1). However, the temporal sequence of different phosphorylation events and the precise function of phosphorylation at different sites need further investigation. Many phosphosites on MCMs identified in vitro also need further identification in cells (Table 2). Current results indicate that the MCM phosphorylation may contribute to its function by affecting MCM complex formation, chromatin binding, and (or) the helicase activity. Phosphorylation of specific sites is likely to trigger detachment of the MCM complex from chromatin. The majority of chromatin loading and phosphorylation events are regulated in a cell cycle-dependent manner. Recently, it has be found that several MCM dephosphorylation events, mediated by Rap1-interacting factor 1 (RIF1)-protein phosphatase 1 (PP1) [49, 68] and phosphatase and tensin homolog deleted on chromosome ten (PTEN) [69], contribute to the strict regulation of DNA replication and replisome stability. Collectively, the phosphorylation and dephosphorylation of MCM proteins regulate cell cycle progression and protect genomic stability, whereas dysfunctional phosphorylation events appear to correlate with the occurrence and development of cancers (Fig. 2). Recently, protein kinase inhibitors targeting essential cell cycle regulators or major signaling pathways have become a major field of investigation for new therapeutic strategies. Drugs targeting MCM phosphorylation may be a potential method for cancer therapy. Evidence has shown that Cdc7-selective inhibitors may decrease MCM2 phosphorylation, inhibit DNA synthesis, and cancer cell viability [70–78]. Further investigation of the functional significance of MCMs phosphorylation in cancer cells is required as it may contribute to the development of novel cancer targets.

**Table 2:** Summary of potential phosphosites on MCMs

Protein	Kinase	Phosphosite	References
MCM2	Cdc7	S31, S220	[43]
	Cdc7	S4, S7	[37]
	Cdk2/CycE1	S13, S27, S381	[37]
	Cdk1, Cdk2	S13, S27, S41	[19]
	SIK1	S7, S27, S41, Y90, S139	[61]
	CK2	S139	[19]
MCM4	Cdk2/CycE1	T94	[37]
MCM7	Cdk2/CycE	S365	[37]

## Abbreviations

ATM: ataxia-telangiectasia-mutated kinase
ATR: ATM- and Rad3-related kinase
Cdc6: cell division cycle 6
Cdc7: cell division cycle 7
Cdc45: cell division cycle 45
Cdk: cyclin-dependent kinase
Cdt1: Cdc10 dependent transcript 1
Chk1: checkpoint kinase 1
CMG: Cdc45–MCMs–GINS
DAPK: death-associated protein kinase
DDK: Dbf4-dependent kinase
DDR: DNA damage response
EBV: Epstein–Barr virus
EBV-PK: EBV-encoded protein kinase
EGFR: epidermal growth factor receptor
GINS: Go, Ichi, Ni, and San
HERC2: HECT and RLD domain containing E3 ubiquitin protein ligase 2
HU: hydroxyurea
ILK: integrin-linked kinase
IMEs: immortalized human diploid mammary epithelials
IMFs: immortalized human fibroblasts
ITGA7: integrin α7
IR: ionizing radiation
MEFs: mouse embryo fibroblasts
NHDF: human dermal fibroblast
ORC: origin recognition complex
PP1: protein phosphatase 1
pre-RCs: pre-replicative complexes
PTEN: phosphatase and tensin homolog deleted on chromosome ten
RACK1: receptor for activated C kinase 1
RIF1: Rap1-interacting factor 1
SIK1: salt-induciblekinase1
